# The impact of global lineage dynamics, border restrictions, and emergence of the B.1.1.7 lineage on the SARS-CoV-2 epidemic in Norway

**DOI:** 10.1093/ve/veab086

**Published:** 2021-09-23

**Authors:** Magnus N Osnes, Kristian Alfsnes, Jon Bråte, Ignacio Garcia, Rasmus K Riis, Kamilla H Instefjord, Hilde Elshaug, Hilde S Vollan, Line Victoria Moen, Benedikte Nevjen Pedersen, Dominique A Caugant, Kathrine Stene-Johansen, Olav Hungnes, Karoline Bragstad, Ola Brynildsrud, Vegard Eldholm

**Affiliations:** Division of Infectious Disease Control and Environmental Health, Norwegian Institute of Public Health, Lovisenberggata 6, Oslo 0456, Norway; Division of Infectious Disease Control and Environmental Health, Norwegian Institute of Public Health, Lovisenberggata 6, Oslo 0456, Norway; Division of Infectious Disease Control and Environmental Health, Norwegian Institute of Public Health, Lovisenberggata 6, Oslo 0456, Norway; Division of Infectious Disease Control and Environmental Health, Norwegian Institute of Public Health, Lovisenberggata 6, Oslo 0456, Norway; Division of Infectious Disease Control and Environmental Health, Norwegian Institute of Public Health, Lovisenberggata 6, Oslo 0456, Norway; Division of Infectious Disease Control and Environmental Health, Norwegian Institute of Public Health, Lovisenberggata 6, Oslo 0456, Norway; Division of Infectious Disease Control and Environmental Health, Norwegian Institute of Public Health, Lovisenberggata 6, Oslo 0456, Norway; Division of Infectious Disease Control and Environmental Health, Norwegian Institute of Public Health, Lovisenberggata 6, Oslo 0456, Norway; Division of Infectious Disease Control and Environmental Health, Norwegian Institute of Public Health, Lovisenberggata 6, Oslo 0456, Norway; Division of Infectious Disease Control and Environmental Health, Norwegian Institute of Public Health, Lovisenberggata 6, Oslo 0456, Norway; Division of Infectious Disease Control and Environmental Health, Norwegian Institute of Public Health, Lovisenberggata 6, Oslo 0456, Norway; Division of Infectious Disease Control and Environmental Health, Norwegian Institute of Public Health, Lovisenberggata 6, Oslo 0456, Norway; Division of Infectious Disease Control and Environmental Health, Norwegian Institute of Public Health, Lovisenberggata 6, Oslo 0456, Norway; Division of Infectious Disease Control and Environmental Health, Norwegian Institute of Public Health, Lovisenberggata 6, Oslo 0456, Norway; Division of Infectious Disease Control and Environmental Health, Norwegian Institute of Public Health, Lovisenberggata 6, Oslo 0456, Norway; Division of Infectious Disease Control and Environmental Health, Norwegian Institute of Public Health, Lovisenberggata 6, Oslo 0456, Norway

**Keywords:** SARS-CoV-2, phylogeography, phylodynamics, transmission, import

## Abstract

As the COVID-19 pandemic swept through an immunologically naïve human population, academics and public health professionals scrambled to establish methods and platforms for genomic surveillance and data sharing. This offered a rare opportunity to study the ecology and evolution of SARS-CoV-2 over the course of the ongoing pandemic. Here, we use population genetic and phylogenetic methodology to characterize the population dynamics of SARS-CoV-2 and reconstruct patterns of virus introductions and local transmission in Norway against this backdrop. The analyses demonstrated that the epidemic in Norway was largely import driven and characterized by the repeated introduction, establishment, and suppression of new transmission lineages. This pattern changed with the arrival of the B.1.1.7 lineage, which was able to establish a stable presence concomitant with the imposition of severe border restrictions.

## 1. Introduction

The COVID-19 pandemic has brought about the rapid development and uptake of genomic epidemiology globally. More than 2 million SARS-CoV-2 sequences have been shared through the GISAID initiative (Elbe and Buckland-Merrett 2017) as of June 2021. Genome sequences sampled across time and space are optimally suited for tracking and making sense of the evolution and spread of pathogens. In the case of SARS-CoV-2, both GISAID and NextStrain (Hadfield et al., 2018) have become essential platforms for tracking the dispersal of viral variants and mutations globally.

A hallmark of the COVID-19 pandemic has been the wave-like regional and global sweeps of new variants. The expansion of some clades has likely been the result of societal factors and travel patterns, such as the spread of the 20E (EU1) clade out of Spain (Hodcroft et al., 2020). Sweeps of other variants have, on the other hand, clearly been the result of increased transmissibility. The most notable examples of the latter include the 20A clade (carrying the D614G mutation), which became fixed in the first half of 2020 (Korber et al., 2020), and the sweep of Pangolin lineage B.1.1.7 (Alpha variant), starting in the autumn of 2020 (Davies et al., 2021). The second half of 2020 was also characterized by parallel regional sweeps of P.1 in South America (Gamma variant) (Faria et al., 2021) and B.1.351 (Beta variant) in Africa (Tegally et al., 2020; O’Toole et al., 2021), both of which being less efficiently inhibited by neutralizing antibodies (Hoffmann et al., 2021). B.1.1.7, B.1.351, and P.1 were all recognized as variants of concern (VOCs) by the European Centre for Disease Prevention and Control (https://www.ecdc.europa.eu/en/covid-19/variants-concern, last accessed on 1 May 2021).

The UK spearheaded efforts to implement large-scale sequencing for the surveillance of SARS-CoV-2 transmission dynamics and evolution. A recent study, capitalizing on the fine-grained genomic data available, relied on time-stamped phylogeographic analyses to shed light on viral lineage dynamics in the UK (du Plessis et al., 2021). From these analyses, the authors were able to quantify introductions over time and assess the effect of lockdowns on importation and transmission rates.

The COVID-19 pandemic in Norway has been shaped by repeated introductions of new viruses (Seppälä et al., 2020). This was evident from the earliest stage of the pandemic, when a large number of infected tourists returned from Lombardy (Brynildsrud and Eldholm 2020) and ski resorts in Austria (Norwegian Institute of Public Health 2020). Here, we compare and contrast the lineage dynamics of SARS-CoV-2 in Norway with those observed in Europe and globally. We also perform phylogeographic analyses to quantify virus introductions and local transmission in Norway. These analyses illuminate how the COVID-19 pandemic in Norway reflects both global lineage dynamics and the effects of non-pharmaceutical interventions, including border restrictions.

## 2. Results

### 2.1 Lineage dynamics in Norway

**Figure 1.**
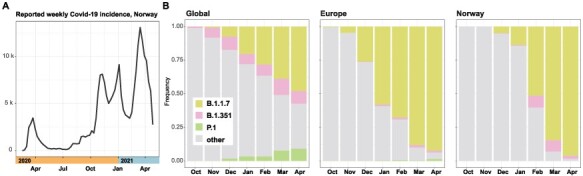
COVID-19 incidence in Norway and lineage dynamics across geographic scales. (A) Weekly reported COVID-19 incidence in Norway from the start of the pandemic until the end of April 2021. (B) Frequencies of VOCs over time across geographic scales. Only variants defined as VOCs by European Centre for Disease Prevention and Control (ECDC) as of 6 May 2021 are highlighted.

The first case of COVID-19 was confirmed in Norway on 26 February 2020 and extensive national control measures were implemented on 12 March (Seppälä et al., 2020). The weekly numbers of reported COVID-19 cases throughout the first ∼13 months of the pandemic in Norway is shown in Fig. 1A. It should be noted that case numbers in the early period are under-reported, as test-criteria were strict to avoid exceeding the total testing capacity. From 12 August 2020, tests have been available for anyone suspecting to be infected with SARS-CoV-2.

To characterize SARS-CoV-2 lineage dynamics in Norway and compare these with larger trends globally and in Europe, we first generated time-structured representative datasets at the levels of ‘Norway’, ‘Europe’, and the world (‘global’) from GISAID. The global dataset included 70 genomes per world region (Europe, Asia, Oceania, Africa, North America, and South America) per month, beginning in March 2020 and ending with April 2021. The European and Norwegian datasets were generated by selecting 400 random genomes per month (see Methods for details).

Following the origin of the COVID-19 pandemic in China, multiple seeding events of SARS-CoV-2 had taken place by the time travel restrictions were put in place across the globe. In Europe, sustained local transmission was ongoing as early as January/February 2020 (Nadeau et al., 2021). To reconstruct the rise of particular lineages of interest, we investigated lineage distributions over time across the three geographic scales. On the NextStrain platform, there are multiple schemes for defining clades and lineages. The Pangolin lineage scheme (github.com/cov-lineages/pangolin) is particularly useful as it is fine-grained and has formed the basis for defining VOCs (see https://www.ecdc.europa.eu/en/covid-19/variants-concern, last accessed 15 April 2021). The analyses were restricted to the period October 2020 to April 2021, as the number of available genomes from Norway was limited prior to the said period. To ease interpretation, only the three VOCs B.1.1.7, B.1.351, and P.1 are annotated in Fig. 1B.

From the figure, it is evident that the three VOCs expanded in parallel, with B.1.1.7 standing out in terms of frequency. In Europe and Norway, B.1.1.7 replaced other lineages almost entirely over a 6-month period. There was also a higher fraction of B.1.351 in Norway than in Europe in general (Fig. 1B), but this was largely the result of a single outbreak and could also to some degree reflect biased sampling as substantial resources were applied to track and sequence isolates suspected to be linked to the outbreak.

**Figure 2.**
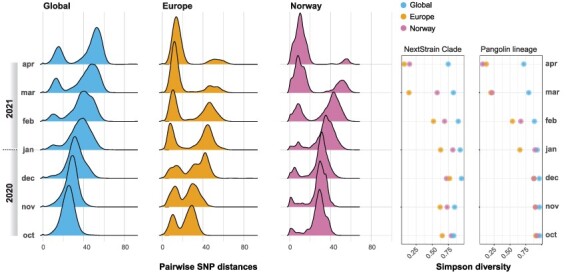
SARS-CoV-2 diversity over time across geographic scales. The ridgeline plots illustrate the evolution of pairwise SNP distances over time (October 2020–April 2021) across subsets of genomes sampled globally in Europe or in Norway only. The dot plots on the right illustrate the corresponding Simpson diversity estimates at the level of NextStrain clades and Pangolin lineages.

Next, we calculated pairwise single nucleotide polymorphism (SNP) distances over time across the three geographic levels. The overall picture is one of selective sweeps occurring over a background of gradually accumulating genetic diversity (Fig. 2). The dynamics are, however, dependent on the geographic scale. The global dataset was mainly characterized by increased diversity over time, with a minor peak containing more closely related sequences. In Europe, on the other hand, the B.1.1.7 sweep was visible as a marked leftward shift in SNP distances over time. The lineage dynamics of B.1.1.7 in Norway mirrored those in Europe in general. The B.1.1.7 sweep was also clearly manifested in the Simpson’s diversity (Simpson 1949) estimated for the same periods, both at the level of NextStrain clades and Pangolin lineages (Fig. 2). Simpson’s diversity captures both the number of different clades/lineages, and their relative abundance, as such encapsulating both richness and evenness. It is also clear that the arrival of the hyper-transmissible B.1.1.7 lineage in Norway was associated with a marked uptick in COVID-19 incidence in February and March (Fig. 1A).

### 2.2 Genomic epidemiology in Norway

**Figure 3.**
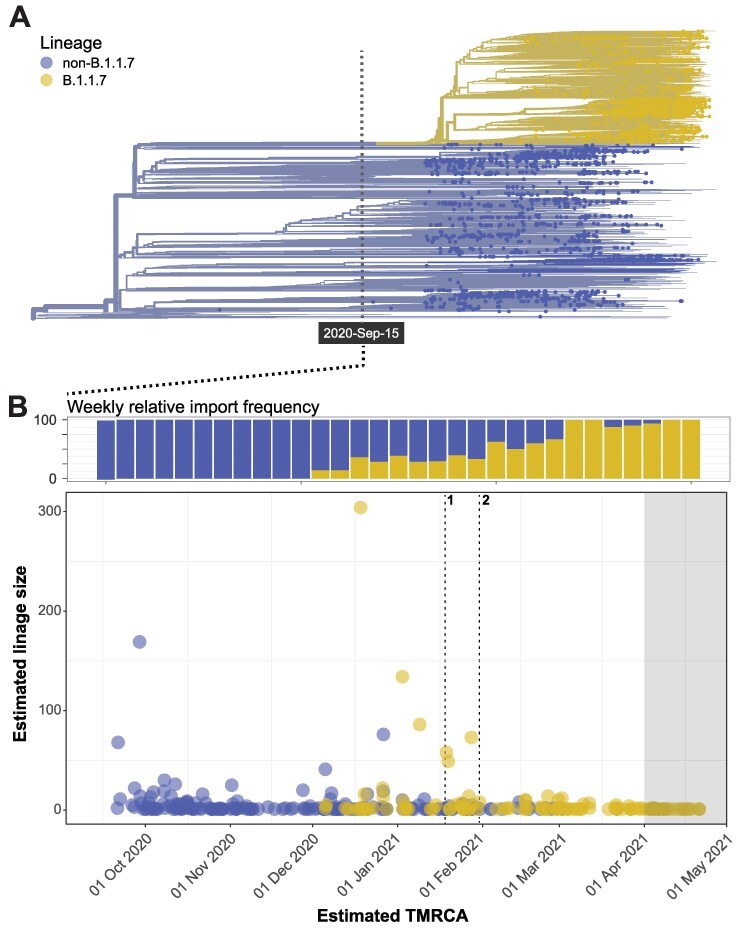
Imports and transmission clusters in Norway. (A) Dated phylogeny generated using the NextStrain pipeline used to infer imports to and local transmission of SARS-CoV-2 in Norway. The B.1.1.7 lineage is colored purple. The last 4 weeks of sampling are shaded grey as there is more uncertainty around these estimates (see main text). B) Weekly relative import frequencies of B.1.1.7 versus non-B.1.1.7 are plotted in the top panel. Individual imports are plotted in the bottom lineage as a function of the estimated TMRCA of each import lineage and the size of the cluster (i.e. the number of sequenced isolates in Norway inferred to be the result of each import). The dashed lines mark the implementation date for key interventions: (1) mandatory testing at the border and (2) closure of the border for individuals not residing in Norway.

Next, we were interested in investigating the interplay between viral variants, importation load, transmissibility, and non-pharmaceutical interventions (NPIs) in shaping the COVID-19 epidemiology in Norway. First, a dated phylogeny was generated, including the 2,544 sequences corresponding to the Norwegian dataset and 5,486 ‘contextual’ sequences from the rest of the world (Fig. 3A; see Methods). Maximum likelihood was used to perform ancestral state analyses, applying a binary categorization of the samples (‘Norway’ and ‘the rest of the world’ [‘RoW’]).

Of the 2,544 Norwegian isolates, 350 (95 per cent CI: 344–357) and 2,194 (95 per cent CI: 2,187–2,200) were inferred to be the result of importation from abroad and from local transmission, respectively. These numbers should not be interpreted to reflect total numbers of imports and local transmissions in Norway as they are inferred from sampled genomes only, but they are still informative as a measure of the relative importance of new introductions for the national epidemic.

The first cases of B.1.1.7 were detected in Norway in December 2020 (Fig. 3B). By mid- February, half of all newly imported cases were caused by B.1.1.7 viruses, reflecting the concomitant sweep of B.1.1.7 in Europe (Fig. 1B). In December 2020 and January 2021, when both B.1.1.7 and non-B.1.1.7 viruses were imported at high frequencies, B.1.1.7 introductions stood out by their ability to generate larger outbreaks in a setting of strict NPIs (Fig. 3B), reflecting the increased transmissibility of the lineage (Davies et al., 2021).

**Figure 4.**
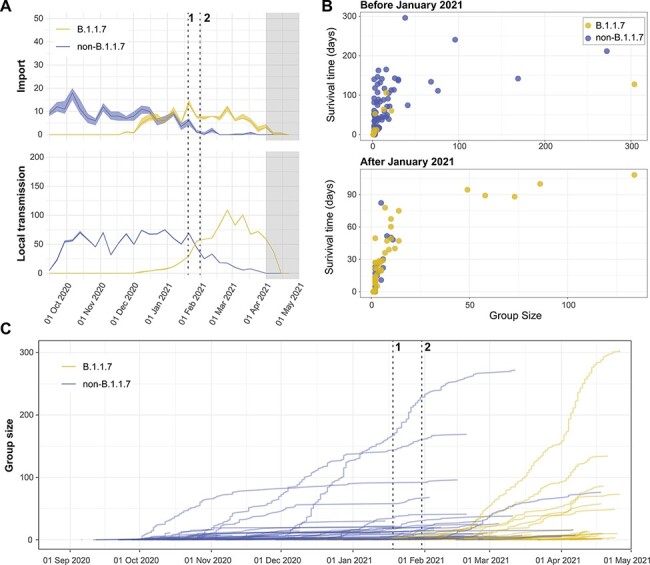
Genome epidemiology of B.1.1.7 and non-B.1.1.7 in Norway. (A) Weekly estimated imports (top panel) and local transmission events (bottom panel) of B.1.1.7 (yellow) and non-B.1.1.7 (blue) in Norway. The dashed lines mark the implementation date for key interventions: (1) mandatory testing at the border and (2) closure of the border for individuals not residing in Norway. The last 4 weeks of sampling are shaded grey as there is more uncertainty around these estimates (see main text). (B) Size and longevity of transmission clusters measured in days. ‘Survival time’ = time (days) between the estimated TMRCA and the last sampled isolate in each transmission lineage (C) Cumulative transmission lineage sizes, where each new case was added to its transmission lineage at the date of sampling. Key interventions are labelled as in (A).

In response to the rapidly increasing COVID-19 incidence during the autumn and subsequent winter of 2020–21 (Fig. 1A), strict border restrictions were implemented in Norway in early 2021. The restrictions were effective in terms of reducing the onward transmission of imported viruses (Fig. 3B) as a higher proportion was detected at the border and/or contained by mandatory quarantine. The number of new introductions per week was also reduced, particularly during the 2 months following the implementation of the border restrictions (Fig. 4A). As a result of strict NPIs, local transmission of non-B.1.1.7 virus was successfully curbed in the following period but, by then, B.1.1.7 was already established in the country and spreading efficiently. By mid-February, the majority of local transmissions in Norway were caused by the B.1.1.7 lineage. In fact, from 1 January 2021, every single import that was able to cause large and lasting outbreaks in Norway belonged to the B.1.1.7 lineage (Fig. 4B,C).

## 3. Discussion

The COVID-19 pandemic is unique in the sense that extensive genome-epidemiological analyses have been performed continuously in near real time. The wealth of sequence data shared and analysed via GISAID (Elbe and Buckland-Merrett 2017) and NextStrain (Hadfield et al., 2018) puts us in a unique position to understand the dynamics of the pandemic in response to both intrinsic viral characteristics, such as the evolution of increased transmissibility, as well as infection control measures, such as travel restrictions and other NPIs.

In the period under study (October 2020–April 2021), the sweep of B.1.1.7, starting in the autumn of 2020 (Davies et al., 2021), is striking. In Europe, B.1.1.7 was first detected in September 2020 and had established complete dominance by April 2021 (Fig. 1B), severely constraining the genomic diversity of circulating viruses on the continent (Fig. 2). In parallel with B.1.1.7, regional sweeps of the P.1 and B.1.351 lineages, harbouring mutations rendering them partially protected from neutralizing antibodies (Hoffmann et al., 2021), occurred in South America and Africa, respectively.

By combining the analyses of global lineage dynamics with ancestral state reconstruction and data on the timing of various interventions, we were able to tease apart various drivers and shapers of the COVID-19 epidemic in Norway. Various NPIs have been in place in Norway from March 2020 to halt the transmission of SARS-CoV-2. Prior to the arrival of B.1.1.7, the epidemic was driven by repeated introductions from abroad, followed by outbreaks that were generally extinguished in short order (see https://covariants.org/per-country). In response to increasing COVID-19 case numbers in Norway and the worsening COVID-19 situation in Europe in the autumn of 2020, strict measures for physical distancing were put in place nationally from 28 October. These restrictions were seemingly successful, and the COVID-19 incidence in Norway was in sharp decline in January and early February of 2021.

In response to early reports of B.1.1.7 being more transmissible (Volz et al., 2021) and possibly more virulent, strict border restrictions were implemented in the second half of January 2021. Numerous rules and regulations were implemented at different time points, but the most drastic were the following: (1) from 18 January, mandatory testing at the border when entering Norway and (2) from 29 January, with a few exceptions, people not residing in Norway were barred from entering the country. For a full timeline of the implementation of interventions, see the Norwegian Government overview (The Norwegian Ministries 2021).

The border restrictions successfully reduced the ability of imported viruses to generate transmission chains in Norway (Figs 3B and 4A). However, by then, multiple B.1.1.7 transmission chains were already established (Fig. 4A,C). As a result, the epidemic in Norway shifted from being largely import driven to a self-sustained epidemic completely dominated by B.1.1.7. The order of events surrounding the B.1.1.7 sweep in Norway closely mirrors the situation in Denmark, where restrictions for travellers from the UK were enforced from 22 December 2020 and general domestic restrictions tightened on 5 January 2021 in order to halt the establishment of B.1.1.7 (Michaelsen et al., 2021). As in Norway, tightened NPIs practically eliminated all other circulating lineages but were insufficient to halt the spread of B.1.1.7. Similarly, in the UK, thousands of B.1.1.7 exports from Greater London to the rest of the country were inferred to have occurred by the time strict NPIs were put in place in Greater London on 20 December 2020 (Kraemer et al., 2021).

Phylogeographic inferences can be strongly impacted by sample collection bias and, even though sampling is rarely perfect, we believe the current study employs a reasonable sampling scheme: the sample size was relatively large (>8,000 genomes) and included both contextual samples (selected for genetic proximity to the Norwegian genomes) and genomes collected randomly from the global collection. Maximum likelihood ancestral state reconstruction provides a quick approach for identifying and estimating the sizes of transmission lineages and for estimating the relative load of importation and local transmission. We developed the package *LineageHomology* (https://github.com/magnusnosnes/LineageHomology) to summarize and visualize ancestral state estimates. In the current study, we included a fixed but limited number of genomes each month. The sampling is thus incomplete, which will almost always be the case in any real-life study. One can, therefore, reasonably expect *LineageHomology* to underestimate the size of local transmission lineages (as in du Plessis et al. (2021)). Similarly, some smaller transmission chains might be mistakenly categorized as singletons or might go undetected due to incomplete sampling. The estimated absolute numbers of imports and transmission lineage sizes will thus be underestimated. The relative sizes of the transmission lineages and estimates of weekly local transmission and importation are, however, expected to be less sensitive to these types of biases and thus provides a valuable assessment of trends in the relative growth of lineages and their influx over time.

*LineageHomology* uses the midpoint of the ancestral branch of the most recent common ancestor (MRCA) as the arrival time of the transmission lineages. For singletons, it uses the midpoint of the ancestral branch of the oldest node that connects to the singleton without a geographical transition. Since detection lag for transmission lineages is strongly size dependent (du Plessis et al., 2021), the estimated arrival times are expected to be more accurate for larger transmission lineages.

The SARS-CoV-2 pandemic is affecting every nation on earth and recent studies have illuminated the role of cross-border transmission in fueling regional spread of the virus. A study from the UK found a surprisingly high prevalence of COVID-19 among people arriving in the country (1.9 per cent in the period between 11 March and 14 April 2021), including all recognized VOCs. The importance of repeated introductions for sustaining national epidemics depends on multiple factors, most prominently the domestic prevalence, the NPIs in place, and the transmissibility of the circulating variants. A phylodynamic study of four island nations found that border restrictions severely restrained the inferred number of new SARS-CoV-2 introductions. Combined with domestic measures to curb human movement, border restrictions were likely pivotal for controlling COVID-19 in these nations (Douglas et al., 2021).

In line with our findings from Norway, a study from Denmark found a substantial epidemiological impact of new introductions. The authors also concluded that the VOC designation of B.1.1.7 and the tightening of domestic NPIs came too late to prevent the lineage from establishing itself in the country (Michaelsen et al., 2021). In Norway, tight border restrictions were enforced in January 2021. These were successful in bringing down the number of new introductions, but by the time they were implemented, multiple introductions of B.1.1.7 to the country had already occurred, and transmission was taking place across multiple chains. It is clear that implementing appropriate interventions early enough as a pandemic plays out is a formidable task. Yet, the current study demonstrates the importance of expediency when enacting policies to halt the transmission of emerging VOCs.

## 4. Materials and methods

### 4.1 Incidence data, Norway

Weekly incidence data for Norway were extracted from the Norwegian Surveillance System for Communicable Diseases (MSIS 2021).

### 4.2 SARS-CoV-2 sequencing

The Norwegian dataset (see next paragraph) included sequences generated at the Norwegian Institute of Public Health using the ARTIC Network nCoV-19 V3 protocol (Quick 2020) and sequences generated at the Norwegian Sequencing Centre NorSeq node using the Swift Amplicon SARS-CoV-2 Panel (Swift Biosciences). Both methods rely on a multiplex PCR strategy. Amplicons generated with the Artic protocol were sequenced on the Nanopore GridION platform (Oxford Nanopore Technologies) or on the Illumina MiSeq platform (Illumina), whereas Swift amplicons were sequenced on the Illumina NovaSeq platform. Consensus genome assemblies were generated using pipelines suited to each library generation approach. The assembly approach for the Arctic amplicons can be found at https://github.com/folkehelseinstituttet/fhi-ncov-seq-pipelines and the assembly pipeline for the Swift amplicons at https://github.com/nsc-norway/covid-seq).

### 4.3 Sequence collection

All viral whole-genome sequences and accompanying metadata were retrieved from GISAID. The sequencing activity in Norway was intensified after the summer of 2020. However, the incidence at the time was low (Fig. 3) and the sequencing activity somewhat biased towards selective sequencing of import cases and specific outbreaks. We, therefore, chose to restrict the comparative analyses across geographical scales (‘global’, ‘Europe’, and ‘Norway’) to the period spanning 1 October 2020 to 30 April 2021. In October (*n* = 286) and November (*n* = 243), all available Norwegian genomes were included, whereas a random selection of 400 genomes was included for the subsequent months. The global and European sample subsets were generated from ∼1.25 million whole-genome sequences available on GISAID as of 6 May as follows: to generate the European dataset, 400 samples were selected at random per month. For the global dataset, 70 genomes were selected per region (Asia, Oceania, Africa, Europe, North America, and South America) resulting in a total of 420 samples included per month. The exception was the last month (April 2021), where only four sequences were available from Africa (Supplementary Table S1). We thus only included 350 genomes in the global dataset for April. The metadata, including identifiers for sequence retrieval, are available as an online resource at https://doi.org/10.6084/m9.figshare.14838843.v1. Sequences were aligned with nextalign 0.2.0 (https://github.com/neherlab/nextalign) using the Wuhan-Hu-1 genome (Wu et al., 2020) as reference.

### 4.4 Diversity estimates and statistics

For each month, the Simpson diversity index was calculated for each geographic level separately using the *diversity* function in the R package *vegan* v.2.5-7 (https://github.com/vegandevs/vegan/). Pairwise SNP distances were calculated for each geographic level per month using snp-dists (https://github.com/tseemann/snp-dists).

### 4.5 Phylogenetic and phylogeographic analyses

The international dataset was created using the Nextstrain pipeline (Hadfield et al., 2018), client version 3.0.3. [some details]. Briefly, we used pre-selected Norwegian isolates as described above and set the Nextstrain workflow to sample up to 2,500 contextual samples based on genetic proximity from the rest of the world. In addition, we allowed up to 4,000 randomly selected non-Norwegian isolates to be included. An isolate was only included if it covered at least 95 per cent of the reference genome Wuhan-Hu-1 (Sequence length >28,500 bp) and had a definite collection date (i.e. a resolution of sampling month was not sufficient). The final dataset included 8,030 genomes. The time-resolved phylogeny was generated with the Nextstrain toolkit ‘augur refine’ command (Huddleston et al., 2021), which uses the program TreeTime (Sagulenko, Puller, and Neher 2018). The following parameters were set: Wuhan-Hu-1 as the root, clock rate set to 0.0008 mutations/site/year (with a standard deviation of 0.0004), skyline coalescent, marginal date-inference, and no covariance when estimating rates. Tips that deviated more than 4 interquartile ranges from the root-to-tip versus time regression were removed. All associated files can be found at https://doi.org/10.6084/m9.figshare.c.5457351.

We used the phylogeny from the Nextstrain build as input for phylogeographic analyses. Geographical locations were divided into two states: ‘Norway’ and RoW and ancestral states were estimated using the R package *ape* v5.4.1 (Paradis and Schliep 2019), implementing a maximum likelihood method (*ace*). We constrained the estimation of the transition matrix by fixing the rate of import to be 10-fold the rate of export. The reasoning behind this choice is as follows: by 10 June 2021, the European Economic Area (EEA) in total has had an approximately 10-fold higher number of deaths per capita than Norway (https://www.ecdc.europa.eu/en/cases-2019-ncov-eueea, last accessed on 1 May 2021), and we can hence reasonably assume that the COVID-19 incidence has been on average around 10 times higher in the EEA in total than in Norway. As incidence data are unreliable in large parts of the world and the EEA, which Norway is part of, is by far the world region most tightly connected to Norway in terms of travel, we used the estimates from the EEA to inform the rate of transitions between Norway and the rest of the world. Thus, we included an a priori assumption that the transition rate was 10 times higher into Norway than out of Norway.

### 4.6 Local transmission lineages and singletons

The R package *LineageHomology* (https://github.com/magnusnosnes/LineageHomology) was used to extract and summarize the output from the reconstructed ancestral geographical locations. *LineageHomology* conceptually mirrors the approach used in a recent study from the UK (du Plessis et al., 2021). It uses the estimated geographical probabilities on each node to define connected groups of taxa (tips in the phylogenetic tree), namely transmission lineages (TL) as in du Plessis et al. (2021). A TL consists of two or more taxa. The probability of the estimated geographical location must be more than 50 per cent for the same geographical location for every node in the tree that links the taxa in the TL, including the tip nodes. A singleton is a taxon that is not connected to any other taxa in the way defined above. If we do not consider sampling bias and unobserved sequences, a TL represents a movement to a different geographical location with subsequent local transmission in that location. A singleton represents a transition to a new geographical location without local transmission. We used the estimated TLs and singletons in Norway to describe the survival time of imported lineages, the size of the TLs over time, and the weekly number of importation and local transmissions. Please see the *LineageHomology* GitHub page for installation instructions and a tutorial.

We defined the survival time of a TL as the time difference between its time to the most recent ancestor (TMRCA) and the latest observed sequence. We assumed that each TL consists of one import event and that the branching points in the TL define local transmissions. We used the midpoint of the edge above its TMRCA as the date of the import of the TLs. For the local transmission events following the import, we used the estimated dates of the phylogenetic branching points in the transmission lineage. Singletons were directly translated to imports, and we set the date of the imports to the midpoint of the edge above the node that defined the transition to the geographical location of the singleton. For recently detected events, there is considerable uncertainty and difference in the sampling probabilities due to lags in the sequencing and uploading of data. As a consequence of this, the classification of taxa to TLs and singletons might change frequently over time for recently observed data. To highlight this uncertainty, we shaded the background of the most recent 4 weeks in the plots.

## Supplementary Material

veab086_Supp

## Data Availability

All the sequence data used in the current work are publicly available, with metadata available as a Figshare project. Developed code is available on GitHub. See main text for details.

## References

[R1] Brynildsrud Ola and Vegard Eldholm (2020), ‘High COVID-19 Incidence among Norwegian Travellers Returned from Lombardy: Implications for Travel Restrictions’, MedRxiv. <https://www.medrxiv.org/content/10.1101/2020.03.20.20038406v1> accessed 13 Jan 2021.

[R2] Davies Nicholas G. et al. (2021) ‘Estimated Transmissibility and Impact of SARS-CoV-2 Lineage B.1.1.7 in England’, *Science*, 372: eabg3055.doi: 10.1126/science.abg3055. PMC8128288 33658326

[R3] Douglas Jordan et al. (2021) ‘Phylodynamics Reveals the Role of Human Travel and Contact Tracing in Controlling the First Wave of COVID-19 in Four Island Nations’, *Virus Evolution*, 7: 1–10.doi: 10.1093/ve/veab052. PMC8344840 34527282

[R4] Elbe Stefan, and Gemma Buckland-Merrett (2017) ‘Data, Disease and Diplomacy: GISAID’s Innovative Contribution to Global Health’, *Global Challenges (Hoboken, NJ)*, 1: 33–46. 31565258 10.1002/gch2.1018 PMC6607375

[R5] Faria Nuno R. et al. (2021) ‘Genomics and Epidemiology of the P.1 SARS-CoV-2 Lineage in Manaus, Brazil’, *Science*, 372: 815–21. 33853970 10.1126/science.abh2644 PMC8139423

[R6] Hadfield James et al. (2018) ‘Nextstrain: Real-Time Tracking of Pathogen Evolution’, *Bioinformatics*, 34: 4121–3. 29790939 10.1093/bioinformatics/bty407 PMC6247931

[R7] Hodcroft Emma B. et al. (2020) ‘Emergence and Spread of a SARS-CoV-2 Variant through Europe in the Summer of 2020’, *MedRxiv : The Preprint Server for Health Sciences*. 10.1101/2020.10.25.20219063.

[R8] Hoffmann Markus et al. (2021) ‘SARS-CoV-2 Variants B.1.351 and P.1 Escape from Neutralizing Antibodies’, *Cell*, 184: 2384–93.e12. 33794143 10.1016/j.cell.2021.03.036 PMC7980144

[R9] Huddleston John et al. (2021) ‘Augur: A Bioinformatics Toolkit for Phylogenetic Analyses of Human Pathogens’, *Journal of Open Source Software*, 6: 2906.doi: 10.21105/joss.02906. PMC8237802 34189396

[R10] Korber Bette et al. (2020) ‘Tracking Changes in SARS-CoV-2 Spike: Evidence that D614G Increases Infectivity of the COVID-19 Virus’, *Cell*, 182: 812–27.e19. 32697968 10.1016/j.cell.2020.06.043 PMC7332439

[R11] Kraemer Moritz U. G. et al. (2021) ‘Spatiotemporal Invasion Dynamics of SARS-CoV-2 Lineage B.1.1.7 Emergence’, *Science*, 373: 889–95. 34301854 10.1126/science.abj0113 PMC9269003

[R12] Michaelsen Thomas Y. et al. (2021) ‘Introduction and Transmission of SARS-CoV-2 B.1.1.7 in Denmark’, *bioRxiv*, MedRxiv.doi: 10.1101/2021.06.04.21258333.

[R13] MSIS (2021), ‘The Norwegian Surveillance System for Communicable Diseases’, MSIS-Statistikk. <https://www.msis.no/> accessed 1 Oct 2021.

[R14] Nadeau Sarah A. et al. (2021) ‘The Origin and Early Spread of SARS-CoV-2 in Europe’, *Proceedings of the National Academy of Sciences of the United States of America*, 118: e2012008118.doi: 10.1073/pnas.2012008118. PMC7936359 33571105

[R15] Norwegian Institute of Public Health (2020), ‘COVID-19 Ukerapport: 12 2020 (13.3–19.3)’. Norwegian Institute of Public Health.

[R17] O’Toole Áine et al. (2021) ‘Tracking the International Spread of SARS-CoV-2 Lineages B.1.1.7 and B.1.351/501Y-V2’, *Wellcome Open Research*, 6: 121. 10.12688/wellcomeopenres.16661.1 PMC8176267 34095513

[R19] Paradis Emmanuel, and Klaus Schliep (2019) ‘Ape 5.0: An Environment for Modern Phylogenetics and Evolutionary Analyses in R’, *Bioinformatics*, 35: 526–8. 30016406 10.1093/bioinformatics/bty633

[R20] du Plessis Louis et al. (2021) ‘Establishment and Lineage Dynamics of the SARS-CoV-2 Epidemic in the UK’, *Science*, 371: 708–12.doi: 10.1126/science.abf2946. PMC7877493 33419936

[R21] Quick Josh (2020), *nCoV-2019 Sequencing Protocol v3 (LoCost)*. <https://www.protocols.io/view/ncov-2019-Sequencing-Protocol-v3-Locost-bh42j8ye?> accessed 25 Aug 2020.

[R22] Sagulenko Pavel, Vadim Puller, and Richard A. Neher (2018) ‘TreeTime: Maximum-Likelihood Phylodynamic Analysis’, *Virus Evolution*, 4: vex042. 10.1093/ve/vex042 PMC5758920 29340210

[R23] Seppälä Elina et al. (2020) ‘COVID-19 Cases Reported to the Norwegian Institute of Public Health in the First Six Weeks of the Epidemic’, *Tidsskrift for Den Norske Laegeforening*, 140. 10.4045/tidsskr.20.0525. 10.4045/tidsskr.20.0525 33322882

[R24] Simpson E. H. (1949) ‘Measurement of Diversity’, *Nature*, 163: 688.

[R25] Tegally Houriiyah et al. (2020) ‘Emergence and Rapid Spread of a New Severe Acute Respiratory Syndrome-Related Coronavirus 2 (SARS-CoV-2) Lineage with Multiple Spike Mutations in South Africa’, *bioRxiv*, MedRxiv. 10.1101/2020.12.21.20248640.

[R16] The Norwegian Ministries (2021), *Timeline: News from Norwegian Ministries about the Coronavirus Disease Covid-19*. <https://www.regjeringen.no/en/topics/koronavirus-covid-19/timeline-for-news-from-norwegian-ministries-about-the-coronavirus-disease-covid-19/id2692402/> accessed 1 May 2021.

[R26] Volz Erik et al. (2021) ‘Transmission of SARS-CoV-2 Lineage B.1.1.7 in England: Insights from Linking Epidemiological and Genetic Data’, *bioRxiv*, MedRxiv. 10.1101/2020.12.30.20249034.

[R27] Wu Fan et al. (2020) ‘A New Coronavirus Associated with Human Respiratory Disease in China’, *Nature*, 579: 265–9. 32015508 10.1038/s41586-020-2008-3 PMC7094943

